# Storage Quality Changes in Craft and Industrial Blueberry, Strawberry, Raspberry and Passion Fruit-Mango Sorbets

**DOI:** 10.3390/foods12142733

**Published:** 2023-07-18

**Authors:** Agnieszka Palka, Aleksandra Wilczyńska

**Affiliations:** Department of Quality Management, Faculty of Management and Quality Science, Gdynia Maritime University, 81-225 Gdynia, Poland

**Keywords:** storage, fruit sorbet, antioxidative activity, craft sorbets, quality, shelf-life

## Abstract

Sorbets are a popular dessert, especially during hot summer days. They can also have health-promoting qualities, mainly due to the nutritional value of the fruit from which they are made. The production technology can also have an impact on the final nutritional quality of the sorbets. This paper presents a comparative assessment of the quality of industrial fruit sorbets and their craft analogs. Sorbets with the following flavors were selected for the research: blueberry, strawberry, raspberry, and passion fruit with mango. An organoleptic evaluation was performed, and the overrun, melting resistance, active acidity (pH), color in the CIE Lab system, antiradical activity (DDPH method), and content of vitamin C and total polyphenols were determined. The research revealed the differences between sorbets produced from different fruits as well as the differences depending on the production method between products made of the same type of fruit. Craft sorbets were found to be better than industrial sorbets, and storage time had a significant effect on the sorbets’ quality. In terms of organoleptic characteristics, craft mango-passion fruit sorbet turned out to be the best; in terms of antioxidant properties, craft raspberry and strawberry sorbets were the best, and these two sorbets also showed good, stable overrun and melting resistance values during storage.

## 1. Introduction

Sorbets are healthy ice cream alternatives and frozen desserts desired by vegetarians and vegans [1,2,3]. The pro-health properties of sorbets result from the fruits used for their production, as well as from the processing technology. The beneficial effects associated with the consumption of fruits and vegetables are generally attributed to their chemical composition, as they provide health-promoting compounds. The antioxidant power of fruit is closely correlated with the presence of effective free radical scavengers, such as vitamin C and phenolic compounds [4,5]. All plants contain phenolic acids. Phenolic acids are related to the color, sensory qualities, nutritional, and antioxidant properties of food [6,7].

Berries have grown in popularity in recent years and are available to eat fresh or processed. Berries are a natural source of antioxidants and have health-promoting properties, including anti-inflammatory and antioxidant properties [8,9,10,11,12]. Compounds such as polyphenols, anthocyanins, quercetin, rutin, vitamins (vitamin C), and carotenoids have a strong positive effect on human health, possessing anti-inflammatory, anti-allergic, and anti-cancer properties due to their significant antioxidant activity [13,14,15,16,17,18,19]. Berry consumption protects against neurodegenerative and cardiovascular diseases, inflammation, and diabetic osteoporosis, due to the increase in bone mineral density of the whole body [13,20,21,22].

Flavonoid content is higher in blueberries and blackberries than in raspberries and strawberries [23]. The most commonly eaten berries are blueberries (*Vaccinium corymbosum* L.), strawberries (*Fragaria* spp.), raspberries (*Rubus idaeus* L.), and blackberries (*Rubus* spp.). Blackberry fruit sorbets can be an attractive carrier of phenolic compounds with health-promoting properties in the diet during periods of limited access to fresh fruit. Consumers want healthy and nutritious food that will help them avoid health risks and improve their health. As a result, customers are increasingly interested in nutritional and functional foods [23,24,25].

Strawberries are a common and important fruit due to their high content of essential nutrients and beneficial phytochemicals, mainly represented by an extensive class of phenolic compounds that perform many non-essential functions in plants and have great biological potential in humans. The phytochemical composition of strawberries depends on maturity, genotype, and storage conditions [4,26]. Strawberries are also important due to their extremely high vitamin C content (58.8 mg per 100 g) [16].

Mango is a tropical fruit rich in polyphenols, but due to its short shelf life it is often processed to preserve the fruit out of season [27,28,29,30]. Passion fruit is an exotic fruit popular for its fruity aroma. The physical and chemical composition of passion fruit varies depending on the variety and environmental factors including climate, soil conditions, and agricultural practices. Passion fruit juice is characterized by an exotic flavor, but also by its significant content of nutrients, including phenolic compounds and ascorbic acid, as well as a lower sugar content [31,32,33,34,35]. Other studies have shown that mango and other exotic fruit sorbets are a good source of phenolic compounds, especially flavonoids [36,37,38].

Freezing is considered to be one of the best methods of food preservation, used especially for seasonal fruit and vegetables. It is recognized as the best post-harvest technique that preserves the flavor of fruit and is the least destructive technology for preserving phenolic compounds in berries, and it is recommended as a pre-treatment for the production of various berry products [5,39]. In addition to the preservation aspects, freezing provides consumers with access to seasonal fruits, such as blueberries, and their preserves throughout the year [39,40]. Freezing is a safe method of preserving the color, taste, and appearance, as well as the antioxidant activity and bioactive compounds of many foods. According to Oliveira et al., strawberry pulp is an important source of nutrients (e.g., vitamin C) and other bioactive compounds (anthocyanins and phenolic compounds). In addition, it retains high antioxidant capacity regardless of the method of pasteurization and freezing, even after 6 months of frozen storage [41]. Ekici et al. suggested that sorbets, which are a traditional Turkish product, can be consumed as functional beverages due to their phenolic content and their antioxidant and anti-radical properties [7].

Vitamin C losses are closely related to post-harvest conditions, including prolonged storage, elevated temperatures, low relative humidity, physical damage to the fruit during handling, and cold injuries. Freezing is widely regarded as a technique that has little detrimental effect on the phenolic content of fruits [40]. According to Oliveira et al., the best conditions for preserving the nutritional value of strawberries are short storage periods not exceeding 180 days at a temperature below −20 °C [41].

Sorbets made on an industrial scale are produced in a different way than craft sorbets. In industrial products, the amount of fruit is lower and the amount of added water and sugar is higher. Craft sorbets are prepared from fresh fruits with lower, or no, addition of stabilizers. Pectin is a natural stabilizer in craft sorbets. Another advantage of craft sorbets is that they are usually consumed fresh; they are stored for two days at the longest in a showcase freezer but can be stored for longer at lower temperatures (below −18 °C)—for half a year or longer. Customers can buy craft ice creams and store them in freezers at home indefinitely. Industrial products are usually stored for months, and the freezing chain is not always provided during distribution.

The research outcomes could be beneficial for sorbet producers and consumers. They demonstrate the differences between craft and industrial sorbets when fresh and during storage. This study can help producers improve the quality of their products, especially due to increasing consumer expectations. Hence, the present study was conducted with four craft and four industrial sorbets, to investigate their quality, and its changes during storage. The aim of the study was to conduct a comparative analysis of the quality of sorbets from four different fruits, produced using two methods (industrial and craft), and changes in quality during storage for 6 months at −25 °C. The aim was achieved through a comparative analysis of sensory features and selected physicochemical characteristics typical of sorbets. In addition, the quality of the products during storage for 6 months was compared, with all analyses carried out every 2 months. The main purpose of the study was to compare the quality of industrial and craft sorbets.

## 2. Materials and Methods

### 2.1. Industrial Sorbets (S)

Industrial sorbets were purchased in a local supermarket. One of the most popular brands in Poland was chosen for the comparative study. Four flavors of sorbets were purchased in the store and transported to a laboratory freezer. The research material consisted of sorbets that were made of selected fruits: (1) blueberry (*Bacca)*, (3) strawberry (*Fragaria × ananassa*), (5) raspberry (*Rubus* L.) and (7) passion fruit (Passiflora edulis) with mango (*Mangifera indica* L.). The composition of purchased products is presented in Table 1. These are very popular products on the ice cream market.

### 2.2. Raw Materials for Craft Sorbets (C)

The basic ingredients of the sorbets were fresh fruits, which were obtained from a local grocery store at edible ripeness. The research material consisted of sorbets that were made of selected fruits: (2) blueberries, (4) strawberries, (6) raspberries and (8) passion fruit with mango. Craft sorbets were made with the addition of water and sucrose. The composition of craft sorbets prepared for the study is presented in Table 2.

### 2.3. Preparation of Sorbets

Sorbets (2), (4), (6), and (8) were made according to the same procedure, each product separately. Blueberries, strawberries and raspberries without calyxes, mango without the stone but with the peel, and passionfruit without the seeds and peel were used. Fruits were chopped and mixed in Thermomix TM5, then water and sugar were added, and the sorbet mixture was mixed in a Thermomix TM5. Each sorbet mixture was mixed separately, stored at 4 °C for 24 h in order to thoroughly combine the ingredients and to swell the pectin (slow mixing was performed during storage), and then frozen in an Ice Cream Machine Unold 48840 (the temperature of the freezer was −20 °C). All sorbets were stored for 6 months at −25 °C in the laboratory of the Gdynia Maritime University and tested every 2 months: after one day (0 months), 60 days (2 months), 120 days (4 months), and 180 days (6 months) of storage. Industrial products were purchased the same week from local supermarkets.

### 2.4. Physico-Chemical Parameters

The melting resistance was measured by determining the number of melted sorbets from a given sample volume. The method consisted in determining the volume as a result of sorbet leachate after 60 min at room temperature. This is an indicator of the resistance of ice cream to melting. Procedure of determination: Cylinder-shaped samples were cut from the frozen ice cream using a metal cylinder-shaped mold with a capacity of 24.73 cm^3^. The entire mass of the mold was transferred to a sieve that was mounted on a funnel. The funnel was placed in a measuring cylinder. After 60 min, the volume of leakage was read from the cylinder. The test was carried out at a temperature of 20 °C [42].

The calculations were obtained according to the formula:

(1) $$V=\frac{V1}{V2}\times 100\%$$

where:

V = melting resistance [%],
V1 = volume of melted sorbet [cm^3^], and
V2 = volume of cylinder (24.73) [cm^3^].

The overrun was measured by determining the amount of air in a given volume of the sample. A defined volume of the sample (cut from the product with a cylinder) was transferred to a volumetric flask and made up to the mark with distilled water. Based on the known volume of the volumetric flask, the volume of the cylinder, and the amount of added water, the overrun of the sorbets was calculated [42].

The total phenolic content (TPC) was determined using Folin–Ciocalteu reagent and a standard curve of gallic acid (AR), and the results were expressed as mg gallic acid equivalent (GAE)/100 g sample [43].

The antioxidant activity was estimated as free radical scavenging activity using 1,1-diphenyl-2-picryl-hydrazil (DPPH) [44].

The vitamin C content was determined by Tillmans’ method [45].

Acidity (pH) was determined according to the IUPAC Recommendations 2002 [46].

All analyses were conducted three times.

### 2.5. Color Analysis

Color measurements of sorbets were carried out using the CIE Lab system. The obtained results were expressed in terms of CIE L*, a*, and b* values. L* indicates brightness, a* represents red to green coordinates, and b* represents the blue to yellow coordinates of a product [20]. The color of the sorbets was determined using a Konica Minolta CM-5 spectrophotometer (Konica Minolta Sensing, Osaka, Japan). Measurements were made at 9 different locations on the surface of the sorbets. Average color parameters were determined, and the Euclidean distance between two points in the three-dimensional space defined by L*, a*, and b* was used to calculate the color difference between samples. The total color differences (1. difference between 0 and 6 months, 2. difference between S and C after 0 months, and 3. difference between S and C after 6 months) were calculated from the formula [47]:

(2) $$\Delta E=\sqrt{\Delta L^{2}+\Delta a^{2}+\Delta b^{2}}$$

where

∆L = brightness difference,
∆a = redness difference, and
∆b = yellowness difference.

In the analysis of the results, a criterion was used according to which absolute color differences (ΔE*) between 0 and 1 are not discernible; those from 1 to 2 are slightly perceptible, recognizable by a person experienced in distinguishing color nuances; those from 2 to 3.5 are recognized even by a non-expert; those from 3.5 to 5 show are clearly seen to be different; and ΔE* above 5 means a large color difference. The above data are statistical, experimentally proven, and commonly used.

### 2.6. Sensory Evaluation

The sensory assessments were performed by 15 trained judges. The coded samples were served to panelists under normal daylight. The temperature of samples was −12 °C (this temperature is recommended when serving ice cream). A 5-point hedonic scale was used for the evaluation of color, odor, taste, consistency, and overall preference of sorbets (Table 3). The sensory assessment was also performed during storage by the same panelists.

### 2.7. Statistical Analysis

The mean and standard deviation were calculated using Statistica v. 13.1 software (StatSoft, Tulsa, OK, USA). One-way and two-way analysis of variance (ANOVA) were conducted to investigate the overall effect of type of sorbet and storage time (months) on the values of individual quality parameters. The Tukey test was used to determine significant differences between means (*p* ˂ 0.05).

## 3. Results and Discussion

The results of the sensory evaluation of craft (C) and industrial (S) sorbets are presented in Table 4. The evaluation of industrial sorbets (S) was made immediately after purchase, and craft sorbets (C) 24 h after production due to the production technology—after freezing, the sorbets were placed in a freezer at −25 °C to provide hardening.

**Figure 1:**
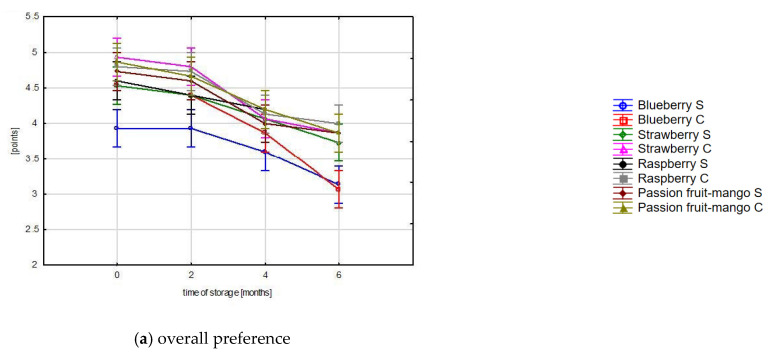

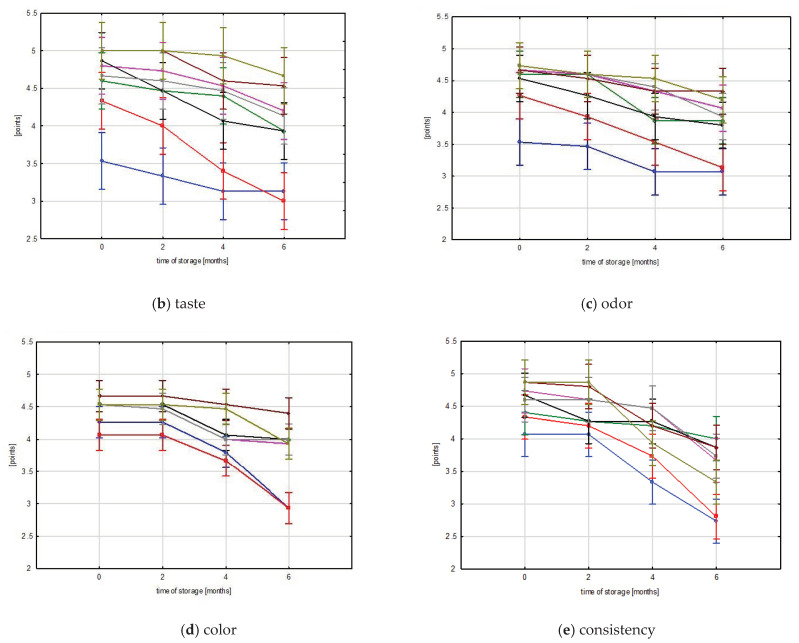
Changes in the scores of the organoleptic features in different sorbet types during storage.

Our study showed that passion fruit-mango sorbets were the best-rated products and received the highest marks in all assessed characteristics. The ratings ranged from 4.5 (odor) to 5.0 (taste), which means that all features were rated very highly. The lowest rated product was blueberry sorbet from a local supermarket. Its characteristics, such as overall preference, consistency, taste, and smell, were rated significantly worse (<4). All other sorbets at the beginning of the research obtained satisfactory sensory evaluation results (above 4). The evaluation of the organoleptic characteristics of the sorbets showed that the quality deteriorated significantly during the six-month storage period (Figure 1).

Storage for 2 months did not lead to significant deterioration in the quality of the tested sorbets, but after that time the changes were very noticeable. The greatest deterioration of the sensory evaluation was found in the case of the consistency of sorbets, most likely as a result of the loss of air from the sorbets and the expansion of ice crystals. These changes occur during the storage of frozen products due to temperature fluctuations [48]. Changes in all sensory characteristics were greater in the case of craft sorbets, which is a natural phenomenon. Craft ice cream and sorbets are produced using a different method than their industrial counterparts and are produced on an ongoing basis in ice cream parlors for direct consumption [49].

Sensory evaluation showed that craft sorbets were of better quality, probably due to their higher fruit content and freshness. Blueberry sorbet bought in a supermarket received the worst rating (3.86), and craft strawberry sorbet was rated the best (4.93). Six months of storage had an impact on all assessed sensory characteristics. At the end of storage, passion fruit and mango sorbets received the highest scores (4.50 (S) and 4.49 (C)).

As the storage progressed, the evaluations of all organoleptic characteristics gradually decreased, with the greatest decrease observed in the case of blueberry sorbet from craft production. It was found that in both blueberry sorbets the evaluation of all characteristics after six months of storage had a score of around three, so their sensory quality was unsatisfactory. In all other sorbets, all distinguishing features, except for consistency, were scored more than four points, which proves good sensory quality of the evaluated products. The analysis of variance showed that the type of sorbet, production methods, and storage method, as well as the combination of these factors, had a significant impact on the sensory quality of sorbets (*p* < 0.05).

The estimation of color differences between sorbets (S) and (C) and color change during storage is shown in Table 5.

It was found that only the color of the craft raspberry sorbet did not undergo any visible changes during 6 months of storage (ΔE = 0.9). A similar, small difference, visible only to an experienced observer (ΔE < 2), was found between passion fruit-mango sorbets (S) and (C) both at the beginning of the experiment and at its end, after 6 months of storage. A color difference visible to an inexperienced observer (2 < ΔE < 3.5) was found between the strawberry sorbets (S) and (C) after 6 months of storage. A similarly visible change in color was found in the passion fruit-mango S sorbet after 6 months of storage, while in the passion fruit-mango C sorbet after 6 months of storage a clear color difference was found (3.5 < ΔE < 5). A similar color difference was found in blueberry and raspberry sorbets depending on the production method (difference between S and C after 0 months) at the beginning of the experiment. Values of ΔE > 5 are marked in red in the table; this indicates a very large difference, representing two different colors.

The results of the assessment of physicochemical parameters of sorbets are presented in Table 6. The values of the examined parameters differed depending on the type of fruit from which they were produced and the production method (*p* < 0.05). Both passion fruit-mango sorbets were characterized by the highest color brightness and the highest value of the b parameter (yellowness). Passion fruit and mango sorbet purchased in a local supermarket had the highest overrun, while strawberry and raspberry sorbets purchased in a local supermarket had the highest resistance to melting.

Craft blueberry and raspberry sorbets had the highest antiradical activity and total phenolic content, and craft strawberry and blueberry sorbets contained the most vitamin C. Passion fruit and mango sorbet purchased at the local supermarket had the lowest antiradical activity and contained little polyphenols and vitamin C.

**Figure 2:**
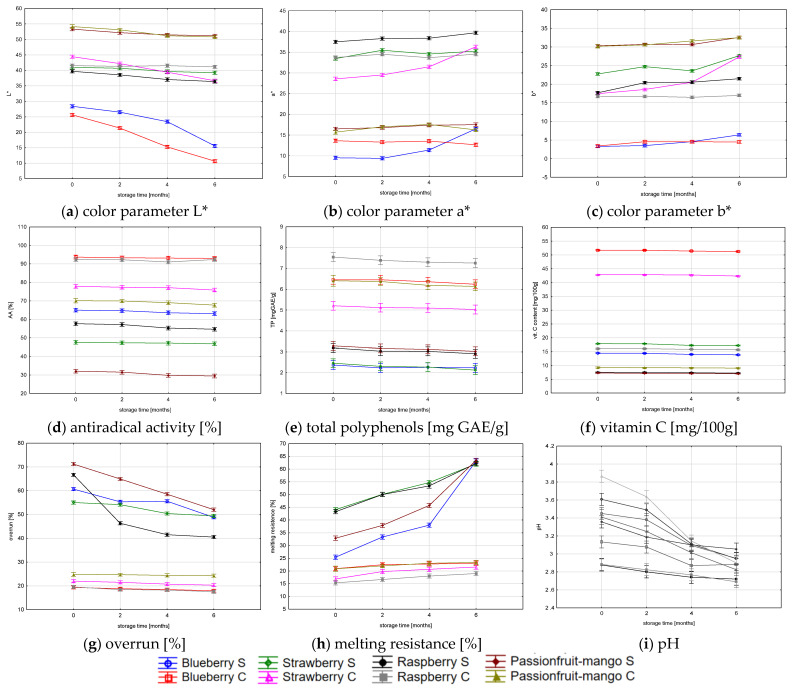
Changes in the values of physicochemical parameters during 6-month storage.

Figure 2 shows changes in the physicochemical characteristics of the tested sorbets during storage. As seen in the charts, during six-month storage, the sorbets darkened, the value of parameter a* (redness) slightly increased, and parameter b* (yellowness) decreased. Their biological activity did not change—total phenolic content, vitamin C content, and antiradical activity remained almost at the same level. It clearly indicates that compounds responsible for antioxidant properties are stable during storage at low temperatures. Similar results were obtained in research conducted by other researchers [50,51,52]. The melting resistance increased and overrun decreased in all sorbets purchased in the local supermarket. The higher overrun of sorbets purchased in the local supermarket was due to the production method. In freezing machines used in artisanal ice cream parlors, the level of product aeration is much lower than in industrial devices [own, unpublished research]. The acidity of all sorbets increased. Changes in pH, overrun, and melting resistance were quite significant, which can be explained, as in the case of changes in organoleptic characteristics, by the loss of air and the growth of ice crystals during storage [53]. During storage, the ice from the sorbet mass sublimated on the surface of the lid of the package in the form of frost, which could have contributed to the decrease in the pH of the sorbet mass. Similar results were obtained in other studies that found a decrease in the water content of ice cream during storage [54]. Therefore, sublimation inhibitors can be used in the production of ice cream subjected to long-term storage [55].

Mango, due to the relatively low water content and high fiber content, is a very good ingredient to use in ice cream because it improves the consistency and is less sensitive to oxidation than berries [26,30,42,56]. Overrun decreased throughout the storage time, which is natural for all frozen desserts due to loss of air put into sorbets during freezing. There were large differences in the overrun of tested sorbets between industrial and craft sorbets because of different production technology. Melting resistance shows the quality of the sorbet mixture used for production, and changes in its value during storage time should not be higher than 50%; in our study, this level was exceeded in all industrial sorbets. This was surprising because in all industrial products one component of the mixture was a stabilizer, which should prevent the sorbet melting too fast. The lower melting resistance values in craft sorbets were probably the result of the high fruit content in all mixtures and the effect of pectin as a natural stabilizer. All fruits used in this experiment are good sources of pectin.

The analysis of variance showed that the storage significantly affected the values of physicochemical parameters (*p* < 0.05).

## 4. Conclusions

Our research indicated that all tested sorbets were of high quality, but craft sorbets were evaluated more highly. Craft sorbets had a higher fruit content and did not contain stabilizers in the mix recipes. It can therefore be concluded that craft sorbets are better than industrial ones. The storage time had a significant impact on the quality of sorbets for both organoleptic and physicochemical properties. Craft sorbets are a better source of vitamin C and polyphenols and have a better anti-radical effect than industrial sorbets. In terms of organoleptic properties, the craft passion fruit-mango sorbet was the best. In terms of antioxidant properties, the craft raspberry and strawberry sorbets turned out to be the best. These two sorbets also showed good, stable values of overrun and resistance to melting during storage. Both industrial and craft sorbets have a good sensory quality for up to 4 months of storage at −25 °C. They retain the best sensory and health-promoting qualities for up to 2 months of storage at this temperature. From our experience, as craft sorbets are produced on an ongoing basis, their quality at the time of purchase is very high. Industrial sorbets have a long shelf life (about 1 year), so their quality after a few months of storage deteriorates. Although they are still edible and safe, this is important from the consumer’s point of view. In our opinion the quality of industrial sorbets can be improved by providing fresh, good quality fruits for production, and reducing the addition of water to the mixtures in favor of the fruit. Potential future research directions suggested by our study include textural and rheological studies, spectrophotometric methods for determining the pectin content of the craft samples, microscopy studies of crystal growth, and correlation analysis between features.

## Figures and Tables

**Table 1:** Composition of industrial sorbets (producer’s information).

No. of Product	Fruits	Composition
1	Blueberry	Water, blueberries (25%), sugar, glucose, glucose syrup, concentrated lemon juice, stabilizers (guar gum, locust bean gum)
3	Strawberry	Water, strawberries (25%), sugar, glucose, glucose syrup, concentrated lemon juice, stabilizers (guar gum, locust bean gum)
5	Raspberry	Water, raspberries (25%), sugar, glucose, glucose syrup, concentrated lemon juice, stabilizers (guar gum, locust bean gum)
7	Mango + Passion fruit	Water, sugar, mango puree (13%), passion fruit juice from concentrated passion fruit juice (12%) (water, concentrated passion fruit juice), glucose syrup, stabilizers (locust bean gum, guar gum), emulsifier (mono- and diglycerides of fatty acids, thickeners (pectin))

**Table 2:** Composition of craft sorbets [%wt].

No. of Product	Fruits	Amount of Fruits [%]	Water [%]	Sucrose [%]
2	Blueberry	50.0	40.0	10.0
4	Strawberry	50.0	40.0	10.0
6	Raspberry	50.0	40.0	10.0
8	Mango + Passion fruit	35.0 + 15.0	40.0	10.0

**Table 3:** A 5-point hedonic scale for sensory evaluation.

Score	Organoleptic Quality				
	Overall Preference	Color	Odor	Taste	Consistency
1	Dislike	Dark	Dislike	Very poor	Very poor
2	Neither like nor dislike	Slightly dark	Neither like nor dislike	Poor	Poor
3	Like slightly	Moderate	Like slightly	Fair	Fair
4	Like moderately	Pale	Like moderately	Good	Good
5	Like very much	Very pale	Like very much	Very good	Very good

Additional information: (1) untypical for used fruits; (2) untypical for used fruits; (3) slightly typical; (4) typical for used fruits; and (5) very typical for used fruits.

**Table 4:** Results of organoleptic evaluation of sorbets at the beginning of the study (month 0).

Type of Sorbet	Overall Preference	Color	Odor	Taste	Consistency
Blueberry S	3.86 ± 0.864 ^d^	4.21 ± 0.579 ^a^	3.57 ± 1.222 ^a^	3.50 ± 1.160 ^a^	4.00 ± 1.038 ^a^
Blueberry C	4.64 ± 0.497 ^a^	4.14 ± 0.535 ^a^	4.14 ± 0.663 ^b^	4.29 ± 0.611 ^b^	4.43 ± 0.756 ^b^
Strawberry S	4.50 ± 0.519 ^a^	4.50 ± 0.519 ^b^	4.57 ± 0.756 ^c^	4.57 ± 0.756 ^c,d^	4.36 ± 0.745 ^b^
Strawberry C	4.93 ± 0.267 ^c^	4.50 ± 0.519 ^b^	4.64 ± 0.497 ^c^	4.79 ± 0.426 ^d^	4.71 ± 0.469 ^d^
Raspberry S	4.57 ± 0.514 ^a^	4.50 ± 0.519 ^b^	4.57 ± 0.514 ^c^	4.86 ± 0.363 ^d^	4.64 ± 0.497 ^d^
Raspberry C	4.79 ± 0.426 ^b^	4.50 ± 0.519 ^b^	4.71 ± 0.469 ^c^	4.64 ± 0.497 ^c,d^	4.57 ± 0.514 ^c^
Passion fruit-mango S	4.71 ± 0.469 ^b^	4.64 ± 0.497 ^b^	4.71 ± 0.469 ^c^	5.00 ± 0.000 ^d^	4.86 ± 0.363 ^e^
Passion fruit-mango C	4.86 ± 0.363 ^b,c^	4.50 ± 0.519 ^b^	4.79 ± 0.426 ^c^	5.00 ± 0.000 ^d^	4.86 ± 0.363 ^e^

Source: own research; results are expressed as mean ± standard deviation. Different letters in columns (a–e) indicate statistically significant differences between mean values at the level *p* ≤ 0.05 according to the Tukey test; S—sorbets purchased in local supermarkets, C—craft sorbets.

**Table 5:** Total color difference: 1. difference between 0 and 6 months, 2. difference between S and C after 0 months, and 3. difference between S and C after 6 months.

	ΔE		
	Difference between 0 and 6 months	Difference between S and C after 0 months	Difference between S and C after 6 months
**Blueberry S**	14.9	5.0	6.5
**Blueberry C**	15.0		
**Strawberry S**	5.5	8.0	2.8
**Strawberry C**	14.8		
**Raspberry S**	5.5	4.2	8.3
**Raspberry C**	0.9		
**Passion fruit-mango S**	3.3	1.4	1.3
**Passion fruit-mango C**	4.4		

Values of ΔE > 5 are marked in red in the table; this indicates a very large difference, representing two different colors.

**Table 6:** Values of sorbets’ physicochemical parameters identified at the beginning of the study (month 0).

Type of Sorbet	Overrun	Melting Resistance	Color Parameters			Antiradical Activity (AA) [%]	Total Polyphenols (TP) [mg GAE/g]	Vitamin C Content [mg/100 g]	pH
			L*	a*	b*				
Blueberry S	60.71 ± 0.576 ^d^	25.41 ± 1.358 ^c^	28.39 ± 0.504 ^a^	9.53 ± 0.280 ^a^	3.28 ± 0.329 ^a^	64.97 ± 0.503 ^c^	2.37 ± 0.177 ^a^	14.48 ± 0.334 ^c^	3.45 ± 0.095 ^c^
Blueberry C	19.32 ± 0.310 ^a^	20.94 ± 0.254 ^a^	25.63 ± 0.138 ^a^	13.65 ± 0.377 ^b^	3.40 ± 0.181 ^a^	93.70 ± 0.200 ^d^	6.46 ± 0.162 ^c^	51.72 ± 0.010 ^e^	3.13 ± 0.021 ^b^
Strawberry S	55.06 ± 0.705 ^c^	44.06 ± 0.949 ^d^	40.99 ± 0.200 ^b^	33.46 ± 0.410 ^d^	22.75 ± 0.115 ^c^	47.63 ± 1.115 ^b^	2.46 ± 0.459 ^a^	17.86 ± 0.092 ^c^	3.61 ± 0.012 ^c^
Strawberry C	21.99 ± 0.350 ^b^	16.87 ± 0.433 ^b^	44.43 ± 0.362 ^b^	28.57 ± 0.232 ^c^	17.45 ± 0.280 ^b^	77.87 ± 0.945 ^e^	5.21 ± 0.159 ^d^	42.82 ± 0.012 ^d^	3.86 ± 0.012 ^d^
Raspberry S	66.71 ± 0.837 ^d^	43.27 ± 1.513 ^d^	39.70 ± 0.266 ^b^	37.50 ± 0.410 ^e^	17.70 ± 0.389 ^b^	57.63 ± 1.115 ^c^	3.19 ± 0.254 ^b^	7.48 ± 0.085 ^a^	2.88 ± 0.040 ^a^
Raspberry C	19.52 ± 0.469 ^a^	15.33 ± 0.577 ^b^	41.54 ± 0.332 ^b^	33.74 ± 0.236 ^d^	16.71 ± 0.059 ^b^	92.37 ± 0.252 ^d^	7.55 ± 0.251 ^e^	16.07 ± 0.010 ^c^	2.88 ± 0.015 ^a^
Passion fruit-mango S	71.24 ± 0.350 ^e^	32.89 ± 0.912 ^c,d^	53.31 ± 0.434 ^c^	16.51 ± 0.416 ^b^	30.31 ± 0.401 ^d^	32.03 ± 0.603 ^f^	3.28 ± 0.180 ^b^	7.27 ± 0.167 ^a^	3.36 ± 0.081 ^c^
Passion fruit-mango C	24.99 ± 0.837 ^b^	20.80 ± 0.254 ^a^	54.46 ± 0.516 ^c^	15.69 ± 0.104 ^b^	30.18 ± 0.080 ^d^	70.23 ± 0.306 ^a^	6.46 ± 0.162 ^c^	9.24 ± 0.127 ^b^	3.42 ± 0.023 ^c^

Source: own research; results are expressed as mean ± standard deviation. Different letters in columns (a–f) show statistically significant differences between mean values at *p* ≤ 0.05 level according to the Tukey test; S—sorbets purchased in local supermarkets, C—craft sorbets.

## Data Availability

Data are contained within the article.

## Sample Availability

Samples of the compounds are available from the authors.
